# The curse of observer experience: Error in noninvasive genetic sampling

**DOI:** 10.1371/journal.pone.0229762

**Published:** 2020-03-13

**Authors:** Jillian M. Soller, David E. Ausband, Micaela Szykman Gunther

**Affiliations:** 1 Department of Wildlife, Humboldt State University, Arcata, California, United States of America; 2 University of Montana Cooperative Wildlife Research Unit, Missoula, Montana, United States of America; Smithsonian Conservation Biology Institute, UNITED STATES

## Abstract

Noninvasive genetic sampling (NGS) is commonly used to study elusive or rare species where direct observation or capture is difficult. Little attention has been paid to the potential effects of observer bias while collecting noninvasive genetic samples in the field, however. Over a period of 7 years, we examined whether different observers (n = 58) and observer experience influenced detection, amplification rates, and correct species identification of 4,836 gray wolf (*Canis lupus*) fecal samples collected in Idaho and Yellowstone National Park, USA and southwestern Alberta, Canada (2008–2014). We compared new observers (n = 33) to experienced observers (n = 25) and hypothesized experience level would increase the overall success of using NGS techniques in the wild. In contrast to our hypothesis, we found that new individuals were better than experienced observers at detecting and collecting wolf scats and correctly identifying wolf scats from other sympatric carnivores present in the study areas. While adequate training of new observers is crucial for the successful use of NGS techniques, attention should also be directed to experienced observers. Observer experience could be a curse because of their potential effects on NGS data quality arising from fatigue, boredom or other factors. The ultimate benefit of an observer to a project is a combination of factors (i.e., field savvy, local knowledge), but project investigators should be aware of the potential negative effects of experience on NGS sampling.

## Introduction

Noninvasive genetic sampling (NGS) has been implemented with increased frequency in population ecology and conservation biology studies in recent years. NGS allows for the study of a wide range of taxa [1–3] without disturbing or influencing the behavior of the study species [3–6]. Without the use of NGS, invasive or lethal means are relied upon for the collection of genetic samples [7]. Recently, biologists have demonstrated a desire to move away from these invasive methods and are instead choosing to use non-invasive techniques when possible.

NGS has been used to study many species successfully, including the elusive gray wolf (*Canis lupus*). Collecting genetic information non-invasively using scat and hair has been effective for long-term population monitoring and detecting occupancy over a large spatial area [8–10]. Extracting and analyzing DNA from wolf scat and hair has been used as a method to determine population size [9,11,12], pack structure [9,12,13], and dispersal patterns [14,15] without the need for direct observation or capture and radiomarking. Collecting an adequate number of samples can be difficult for species at low densities, however, and may require the collection of hundreds of samples to ensure adequate detection rates [10,12]. Social species, such as the gray wolf, allow for the opportunity to collect many samples of multiple individuals in the same location. In summer, wolves use rendezvous sites, which are areas where members of a pack congregate and provision relatively stationary pups for several weeks until the pups are old enough to travel with the adults in the group [16]. Sampling efforts can be concentrated in and around these rendezvous sites, where presence is easiest to detect and scat samples representing each member of the pack can be found [8]. The successful collection of fecal samples relies on two main factors, 1) the observer’s ability to correctly identify wolf sign, and 2) adequately sampling feces to yield a consensus genotype.

While NGS has proven to be extremely useful, issues such as allelic dropout and observer bias continue to resurface, though the impacts of these reoccurring issues can be minimized over time with further examination [17]. Our study attempts to shed more light on one of the most common yet difficult issues to address that continue to resurface in studies involving NGS: observer bias. Due to the high frequency of genotypic errors typically associated with NGS [18], minimizing potential sources of error such as observer bias is critical. Some studies have examined this in scat detection for group surveys, but fewer have compared successful data collection between individual observers [19]. Errors associated with fecal genetic sampling in the field include incorrect species identification [20], improper sampling technique, and poor tracking ability yielding low detection rates. Studies have documented inexperienced participants to be a significant source of bias in the field but showing a decrease in error rates over time [21]. In a study involving multiple observers, it is important that error is acknowledged and assessed to maximize data accuracy and efficiency.

We evaluated NGS success rates for observers with varying experience levels to test how experience influenced the ability to collect viable DNA from fecal samples of a target species. We hypothesized that having previous experience on a project and familiarity with project protocols would positively affect an observer’s ability to find and sample wolf scats effectively. Specifically, we predicted that experienced observers would find more wolf fecal samples, have higher DNA amplification rates of those samples, and be more likely to correctly identify wolf scats from other sympatric carnivores (i.e., coyotes, *Canis latrans*; mountain lions, *Felis concolor;* black bears, *Ursus americanus*) compared to new observers.

## Study area

Trained observers collected samples in three main study areas, central Idaho (45.9500, -115.5349), Yellowstone National Park, WY (44.4280, -110.5885), and southwestern Alberta, Canada (49.2478, -114.0902) during June-August, 2008–2014. Observers sampled central Idaho (6,413 km^2^) annually during 2008–2014. Average annual precipitation in central Idaho typically ranged 420–710 mm with average summer daily temperatures between 4–31° C. Common vegetation generally consisted of ponderosa pine (*Pinus ponderosa*), lodgepole pine (*P*. *contorta*), and spruce (*Picea englemannii*) forests, and sagebrush (*Artemesia tridentata*) steppe [22].

Observers collected scats in Yellowstone National Park (8,983 km^2^) during 2012–2014. Yellowstone has average temperatures of 5–27°C and average precipitation between 33–53 mm during the summer months. Yellowstone National Park is dominated by lodgepole pine (*P*. *contorta*) forests and expansive meadow systems.

Observers collected samples in the Canadian Rocky Mountains of southwest Alberta, Canada from 2012–2014. We surveyed sites within an area of 12,950 km^2^ between the United States border and Highway 1 and west of Highways 6 and 22, including an area known as Porcupine Hills. Some grazing lease lands could not be surveyed because we were denied access by leaseholders. The western portion of the southwestern Alberta study area was mountainous and dominated by Douglas fir (*Pseudotsuga menziesii*), lodgepole pine, and spruce mixed forests, that transitioned through aspen (*Populus tremuloides*) to agricultural land and fescue (*Festuca spp*.) dominated grassland in the east [23].

## Methods

### Field methods

We employed paid observers and considered observers who had not been previously employed on the project as “new” and those who had familiarity with the sampling protocols and had worked on the project in previous years as “experienced”. All observers were enrolled in, or recent graduates of, a natural resources program at a university. All observers had prior wildlife-related field experience, although not necessarily using NGS. We trained all observers in wolf sign identification and field navigation for three days prior to sampling. One day included presentations showing pictures of various forms of wolf sign (i.e., tracks, scats, trails), measuring exercises of wolf tracks using plaster molds, and observation of various wolf scat conditions in the lab. We spent two additional days in the field showing observers wolf and coyote as well as other sympatric carnivore tracks and scat. Each observer sampled wolf scats under the supervision of project staff during training.

Observers conducted surveys for wolves at predicted rendezvous sites between mid-June and late August. To target survey efforts, we used a habitat model emphasizing wet meadows and areas with standing water as being consistent locations for rendezvous sites [10]. Observers surveyed sites at dawn and dusk, when wolves are active and more likely to respond to human-mimicked howls [16]. Each survey consisted of driving and walking roads and hiking off trail looking for tracks and scat in and around predicted rendezvous sites. Upon reaching a site, observers used howling to detect the immediate presence of wolves [24]. If there was no howl response from wolves, two observers separated for 30–45 minutes attempting to locate a rendezvous site or wolf sign. If there was a howl response, observers estimated a count of individual wolves and all observers on the crew (up to 6) attempted to locate the activity center, where the majority of sign is concentrated [24]. We made no attempt to sample sites according to experience level (i.e., experienced observes were not assigned more difficult areas to collect scats). We defined scats collected by a single observer outside of an active rendezvous site (>500m from the activity center) as “incidental”.

Observers collected canid scats >2.5 cm in diameter and labeled them as “wolf” in the field [25]. Observers only collected scats believed to be deposited after the most recent winter to try and eliminate the collection of samples left by individuals that may have been harvested during the winter. For each scat, observers removed a small portion of the outer layer of fecal material from the side of the scat with sterilized tweezers [8] and stored them in a 2-ml vial containing DETs buffer [26].

During 2011–2014, wolves in Idaho and Yellowstone National Park were fitted with GPS collars, which allowed for crews to hike directly into active rendezvous sites, leaving little room for the collection of scats from non-target species. We did not include any scat samples collected in Idaho and Yellowstone National Park during these years in our analyses of species identification success rates.

Fieldwork was conducted under the University of Montana’s Institutional Animal Care and Use Committee (IACUC) protocol IACUC 008-09MMMCWRU-031009, and 001-15MMMCWRU-011315. Four wolves were collared under the IACUC protocol numbers listed above. Additional wolves were collared as part of state and tribal wildlife agency monitoring programs (see [27] for additional details), following recommended American Society of Mammalogists guidelines, and the subsequent locations of radio collared wolves were shared with the authors. Our survey locations were on public land (Canadian Crown land and United States Forest Service land).

### Lab and statistical analyses

We sent collected genetic samples to the Laboratory for Ecological, Evolutionary and Conservation Genetics (LEECG) at the University of Idaho, USA, for DNA extraction and amplification. The LEECG extracted DNA from scat samples using Qiagen kits (Qiagen Inc., Valencia, CA) and included a negative control to test for contamination [8]. They first screened all samples with a species-identification test (SpID) using a 3-primer mitochondrial DNA (mtDNA) control region polymerase chain reaction (PCR) [28–31] to remove non-target species (e.g. coyote) and low-quality samples [14]. They attempted to genotype all remaining samples identified as wolf/dog using 9 nuclear DNA microsatellite loci as described in Stansbury et al. (2014) and analyzed an additional 10 loci on samples matching at all but one locus. For genotyping, LEECG initially amplified all samples twice, and required successful amplification of alleles at ≥5 loci for the sample to continue for an additional 1–3 replications; samples that amplified at <5 loci were discarded. For each locus they required ≥2 independent PCR amplifications for consensus of a heterozygote and ≥3 independent PCR amplifications for consensus of a homozygote. LEECG included a negative control in all PCRs to test for contamination. Given the potentially large number of first-degree relatives in our dataset, they required ≥7 loci to consider a sample successfully genotyped and ≥8 loci (P_(ID)_sibs ranging from 0.0004 to 0.0012 across study areas) [32] to confirm multiple detections of the same individual. LEECG compared all consensus genotypes and all unique genotypes of previously identified individuals using GENALEX [33] to match samples and distinguish unique genotypes. They performed a second species confirmation analysis on all unique genotypes in program STRUCTURE v2.3.3 [34] at K = 3 under the general admixture model, with a burn-in of 100,000, and 500,000 Markov Chain Monte Carlo (MCMC) repetitions and 10 iterations to estimate individual ancestry and remove highly probable dogs or coyotes from the dataset. To avoid overestimation and account for undetected genotyping errors, they grouped samples mismatching by allelic dropout at only one locus (e.g. 102, 102 vs. 102, 106) as a single individual [35]. We used RELIOTYPE [36] to test the accuracy of unique genotypes represented by only one noninvasive sample (i.e. single detections) by ensuring the genotype attained a 95% accuracy threshold.

We calculated successful DNA amplification frequency using the samples collected within active rendezvous sites, where scats of non-target species are unlikely. Scats collected outside of active rendezvous sites (incidental samples), which had the potential to be from non-target species, were used to calculate correct (i.e., wolf) species identification frequency. Samples that were collected but failed to amplify usable DNA, and therefore could not be identified to species, were labeled as “failures”.

We used a t-test to assess whether new and experienced observers differed in, 1) the number of scat samples collected, and 2) the number of scats samples collected at active rendezvous sites that successfully amplified (i.e., yielded a consensus genotype). For incidental samples, correctly identifying target species scat is a binary process. We had adequate sample size to use a mixed effects logistic regression model (GLMM) to test for differences in the probability of correct species identification as a function of experience level. We treated year and observer name as random effects and used the “lme4” package in Program R. We considered differences significant when p<0.05.

## Results

Thirty-four individuals participated in the collection of samples. Sixteen individuals participated in multiple years, beginning as a “new” observer and becoming “experienced” observers in subsequent years; ultimately resulting in 58 observer/years (33 new and 25 experienced; 1 observer was the author and was defined as “experienced”).

**Fig 1:**
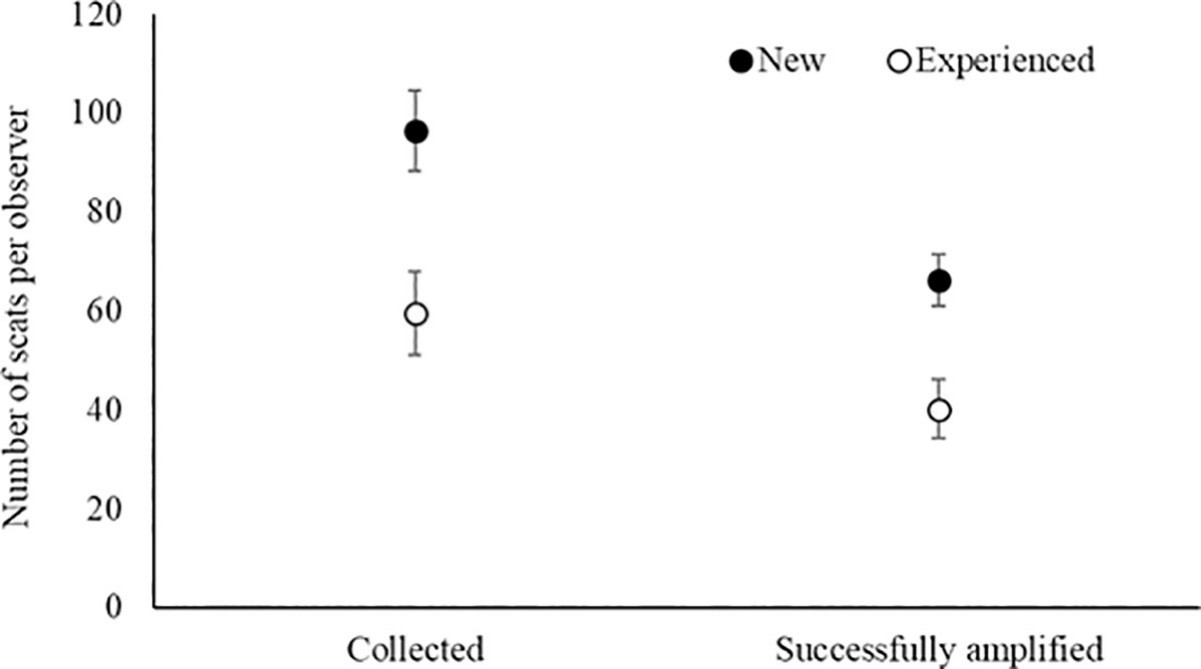
Number of fecal samples collected and successfully amplified as a function of observer experience level (n = 58), during genetic surveys for wolves in Alberta, Canada, central Idaho, and Yellowstone National Park, WY, USA, 2008–2014. Error bars represent the SE.

**Fig 2:**
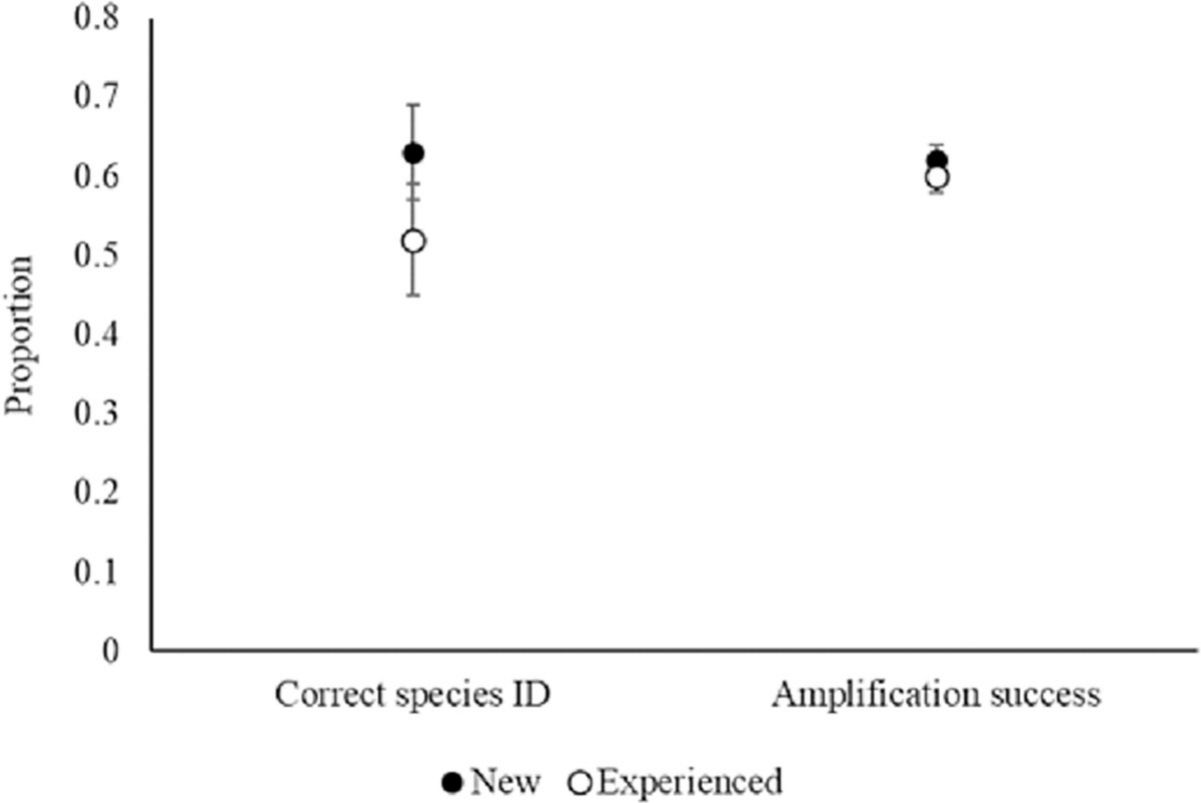
Proportions of rendezvous site fecal samples that successfully amplified and proportions of incidental fecal samples that were correctly identified in the field as wolf as a function of observer experience level (n = 58), during genetic surveys in Alberta, Canada, central Idaho, and Yellowstone National Park, WY, USA, 2008–2014. Error bars represent the SE.

We collected and analyzed 4,836 scat samples, 1,899 (39%) of which failed to yield amplifiable DNA. New observers collected more samples than experienced observers at active wolf rendezvous sites (96.4 vs. 66.2 samples; t = 2.56, df = 54, p = 0.01; Fig 1). New observers also collected more scats than experienced observers that successfully amplified (59.5 vs. 40.2, t = 2.40, df = 52, p = 0.02; Fig 1. The proportion of scats that successfully amplified did not differ between the two observer groups (0.60, SD = 0.13 vs 0.62, SD = 0.11; Fig 2).

Ultimately, when sampling scats that had the potential to be of non-target species (i.e., incidentals), new and experienced observers did not differ in the total number of incidental scats collected (15.1, SD = 7.2 vs. 16.0, SD = 5.8). New observers, however, had a higher probability (0.66 vs 0.50) than experienced observers of correctly identifying the target species from incidental scats. This trend was statistically weaker than other differences we documented (β(experience) = -0.68, p = 0.08; (Fig 2).

## Discussion

Contrary to what we hypothesized, we found that previous experience did not yield better overall results during noninvasive genetic scat sampling. New observers collected more samples and identified the correct species slightly more often than experienced observers. Studies have found that more intensive training protocols decreased error rates of inexperienced participants [37], however our findings suggest that while new observers need rigorous training initially, experienced observers may also need additional attention during (re)training.

We posit two explanations for why experienced observers performed more poorly than expected. First, when sampling incidental scats, experienced observers may not be as tentative as new observers and thus collect more “questionable” scats, which would result in more non-target samples collected and therefore poorer identification rates. Collecting questionable scats (i.e., those that may not be the target species) may be beneficial when surveying for rare or low-density species, because it could decrease the chance an animal goes undetected. Alternatively, collecting scats of non-target species can waste time and resources, potentially impacting the efficiency of the study. Generally, for our study, sampling non-target species was undesirable. Second, experienced observers may simply have been fatigued or bored and thus performed more poorly than new observers. Boredom and fatigue have been known to influence observer performance in wildlife surveys [38], but were not examined in this particular study.

Incidental samples represented 24% of all samples collected but provided critical information about observer effectiveness. Species identification skills, as well as the ability to detect tracks and scats on a road or trail, are crucial during noninvasive sample collection. Experienced observers did not perform better than new observers in this regard, which suggests that while tracking skills and knowledge of the target species’ habits are important, the temperament of human observers also plays an important role in sampling success [19,38]. The majority (76%) of our fecal samples were collected at active wolf rendezvous sites, where accurate species identification of samples is much less critical, as nearly all scats present are wolf. However, such samples are ideal for examining an observer’s collection technique, as the samples are usually in good condition for DNA collection (recently deposited) and abundant.

The effect of individual variation in fatigue, boredom, visual acuity, and experience on sample success are difficult to evaluate [39]. These factors have the potential to bias results especially when implementing the same individual observers over time. The relationship between experience level and both amplification rates and species identification of incidental scats should be considered when implementing noninvasive DNA collection techniques.

DNA amplification rates did not differ between the two observer groups which suggests project managers may want to emphasize other aspects of noninvasive fecal DNA projects (e.g. tracking ability) during hiring and training of new individuals. However, the large range in individuals’ amplification rates (0.29 to 0.89) and correct species identification rates (0.20 to 0.90) that we observed in our study suggests that biologists should consider the variability in success when involving multiple observers in fecal DNA collection. Jenkins and Manly (2008) also found a potential for high variation in detection rates in fecal surveys. The fact that such differences are, in part, reflective of an observer’s experience level suggests that projects using NGS would benefit by working to limit observer bias.

We used genetics to ensure that our target species was correctly identified. Some projects, however, may not have resources for genetic analyses and thus rely solely on observer field identification of target species scat. If observer experience can negatively affect NGS survey success, studies relying on sign surveys and non-genetic approaches may discover ecological consequences associated with observer experience and error. For example, new and experienced observers collected equal numbers of incidental scats, but experienced observers misidentified the target species more often. If we had not verified species identification of incidental samples, genetic sampling conducted by experienced observers would have yielded false positive detections and biased occupancy high because of species misidentification. For rare or imperiled species, such errors can have grave consequences.

An intensive 2-day training period in the field was implemented in this study and could be replicated by others. We show that more seasons spent collecting fecal DNA did not appreciably improve sampling success, therefore the benefits of spending resources to increase in-field training >2 days may be marginal. We recommend carefully weighing observer experience against project goals (e.g., need to detect every individual) and assessing the potential for wasting resources identifying non-target species or, in contrast, missing individuals that may be present. Ultimately, an observer’s contribution to a project relies on a combination of factors (i.e., field savvy, local knowledge) and project investigators should be aware that previous experience collecting fecal genetic samples does not ensure greater success.

## References

[1] Barbosa S, Pauperio J, Searle JB, Alves PC. Genetic identification of Iberian rodent species using both mitochondrial and nuclear loci: application to noninvasive sampling. Molecular Ecology Resources. 2013;13: 43–56. 10.1111/1755-0998.12024 23095787

[2] De Matteo K E, Rinas MA, Argüelles CF, Zurano JP, Selleski N, Di Bitetti MS, et al Noninvasive techniques provide novel insights for the elusive bush dog (*Speothos venaticus*). Wildlife Society Bulletin. 2014;38: 862–873.

[3] Silva TL, Godinho R, Castro D, Abáigar T, Brito JC, Alves PC. Genetic identification of endangered North African ungulates using noninvasive sampling. Molecular Ecology Resources. 2015;15: 652–661. 10.1111/1755-0998.12335 25256349

[4] Wultsch C, Waits LP, Kelly MJ. Noninvasive individual and species identification of jaguars (*Panthera onca*), pumas (*Puma concolor*) and ocelots (*Leopardus pardalis*) in Belize, Central America using cross-species microsatellites and faecal DNA. Molecular Ecology Resources. 2014;14: 1171–1182. 10.1111/1755-0998.12266 24751217

[5] Sugimoto T, Gray TNE, Higashi S, Prum S. Examining genetic diversity and identifying polymorphic microsatellite markers for noninvasive genetic sampling of the Indochinese leopard (*Panthera pardus delacouri*). Mammalian Biology. 2014a;79: 406–408.

[6] Sugimoto T, Aramilev V, Kerley L, Nagata J, Miquelle D, McCullough D. Noninvasive genetic analyses for estimating population size and genetic diversity of the remaining Far Eastern leopard (*Panthera pardus orientalis*) population. Conservation Genetics. 2014b;15: 521–532.

[7] Taberlet P, Luikart G. Non-invasive genetic sampling and individual identification. Biological Journal of the Linnean Society. 1999;68:41–55.

[8] Stenglein JL, De Barba M, Ausband DE, Waits LP. Impacts of sampling location within a faeces on DNA quality in two carnivore species. Molecular Ecology Resources. 2010;10: 109–114. 10.1111/j.1755-0998.2009.02670.x 21564995

[9] Stansbury CR, Ausband DE, Zager P, Mack CM, Miller CR, Pennel MW, et al long-term population monitoring approach for a wide-ranging carnivore: Noninvasive genetic sampling of gray wolf rendezvous sites in Idaho, USA. Journal of Wildlife Management. 2014;78: 1040–1049.

[10] Ausband DE, Mitchell MS, Doherty K, Zager P, Mack CM, Holyan J. Surveying predicted rendezvous sites to monitor gray wolf populations. Journal of Wildlife Management. 2010;74: 1043–1049.

[11] Marucco F, Pletscher DH, Boitani L, Schwartz MK, Pilgrim KL, Lebreton JD. Wolf survival and population trend using non-invasive capture-recapture techniques in the Western Alps. Journal of Applied Ecology. 2009;46: 1003–1010.

[12] Stenglein JL, Waits LP, Ausband DE, Zager P, Mack CM. Estimating gray wolf pack size and family relationships using noninvasive genetic sampling at rendezvous sites. Journal of Mammalogy. 2011;92: 784–795.

[13] Caniglia R, Fabbri E, Galaverni M, Milanesi P, Randi E. Noninvasive sampling and genetic variability, pack structure, and dynamics in an expanding wolf population. Journal of Mammalogy. 2014;95: 41–59.

[14] Lucchini V, Fabbri E, Marucco F, Ricci S, Boitani L, Randi E. Noninvasive molecular tracking of colonizing wolf (*Canis lupus*) packs in the western Italian Alps. Molecular Ecology. 2002;11: 857–868. 10.1046/j.1365-294x.2002.01489.x 11975702

[15] Schwartz MK, Luikart G, Waples RS. Genetic monitoring as a promising tool for conservation and management. Trends in Ecology & Evolution. 2007;22: 25–33. 16962204 10.1016/j.tree.2006.08.009

[16] Mech DL, Boitani L. Wolves: Behavior, ecology, and conservation. Chicago, Illinois: University of Chicago Press; 2003.

[17] Taberlet P, Waits LP, Luikart G. Noninvasive genetic sampling: look before you leap. Trends in Ecology and Evolution. 1999;14(8):323–327. 10.1016/s0169-5347(99)01637-7 10407432

[18] Smith O, Wang J. When can noninvasive samples provide sufficient information in conservation genetics studies? Molecular Ecology Resources. 2014;14: 1011–1023. 10.1111/1755-0998.12250 24620908

[19] Jenkins KJ, Manly BFJ. A double-observer method for reducing bias in faecal pellet surveys of forest ungulates. Journal of Applied Ecology. 2008;45: 1339–1348.

[20] Spiering PA, Gunther MS, Wildt DE, Somers MJ, Maldonado JE. Sampling error in non-invasive genetic analyses of an endangered social carnivore. Conservation Genetics. 2009;10: 2005–2007.

[21] Schmeller DS, Henry P, Julliard R, Gruber B, Clobert J, Dziock F, et al Advantages of volunteer-based biodiversity monitoring in Europe. Conservation Biology. 2009;23: 307–316. 10.1111/j.1523-1739.2008.01125.x 19183201

[22] Western Regional Climate Center. Accessed 10 April 2014. Historical climate information. <http://www.wrcc.dri.edu>

[23] Downing DJ, Pettapiece WW. Natural regions and subregions of Alberta. Natural Regions Committee. Government of Alberta. 2006. Pub. No. I/005. Edmonton, Alberta, Canada.

[24] Harrington FH, Mech LD. Fall and winter homesites use by wolves in northeastern Minnesota. Canadian Field-Naturalist. 1982;96: 79–84.

[25] Weaver JL, Fritts SH. Comparison of coyote and wolf scat diameters. Journal of Wildlife Management. 1979;43: 786–788.

[26] Frantzen MA, Silk JJB, Ferguson JWH, Wayne RK, Kohn MH. Empirical evaluation of preservation methods for faecal DNA. Molecular Ecology. 1998;7: 1423–1428. 10.1046/j.1365-294x.1998.00449.x 9787450

[27] Jimenez MD, Bangs EE, Boyd DK, Smith DW, Becker SA, Ausband DE, et al Wolf dispersal in the Rocky Mountains, Western United States: 1993–2008. 2017;81: 581–592.

[28] Murphy MA, Waits LP, Kendall KC. Quantitative evaluation of fecal drying methods for brown bear DNA analysis. Wildlife Society Bulletin. 2000;28: 951–957.

[29] Dalén L, Götherström A, Angerbjörn A. Identifying species from pieces of faeces. Conservation Genetics. 2004;5: 109–111.

[30] Onorato D, White C, Zager P, Waits LP. Detection of predator presence at elk mortality sites using mtDNA analysis of hair and scat samples. Wildlife Society Bulletin. 2006;34: 815–820

[31] De barba M, Adams JR, Goldberg CS, Stansbury CR, Arias D, Cisneros R, et al Molecular species identification for multiple carnivores. Conservation Genetics Resources. 2014;6: 821–824.

[32] Waits LP, Luikart G, Taberlet P. Estimating the probability of identity among genotypes in natural populations: cautions and guidelines. Molecular Ecology. 2001;10: 249–56. 10.1046/j.1365-294x.2001.01185.x 11251803

[33] Peakall R, Smouse PE. GENALEX 6: genetic analysis in Excel. Population genetic software for teaching and research. Molecular Ecology. 2006;6: 288–295. 10.1093/bioinformatics/bts460 PMC3463245 22820204

[34] Pritchard JK, Stephens M, Donnelly P. Inference of population structure using multilocus genotype data. Genetics. 2000;155: 945–959. 10835412 10.1093/genetics/155.2.945 PMC1461096

[35] Adams JR, Waits LP. An efficient method for screening faecal DNA genotypes and detecting new individuals and hybrids in the red wolf (*Canis rufus*) experimental population area. Conservation Genetics. 2007;8: 123–131.

[36] Miller CR, Joyce P, Waits LP. Assessing allelic dropout and genotype reliability using maximum likelihood. Genetics. 2002;160: 357–366. 11805071 10.1093/genetics/160.1.357 PMC1461941

[37] Fitzpatrick MC, Preisser EL, Ellison AM, Elkinton JS. Observer bias and the detection of low-density populations. Ecological Applications. 2009;19: 1673–1679. 10.1890/09-0265.1 19831062

[38] Norton-Griffiths M. Further aspects of bias in aerial census of large mammals. Journal of Wildlife Management. 1976;40: 368–371.

[39] Neff DJ. The pellet-group count technique for big game trend, census, and distribution: A review. Journal of Wildlife Management. 1968;32: 597–614.

